# Supplementary material for: Baseline knowledge and attitudes on COVID-19 among hotels’ staff: A cross-sectional study in Kigali, Rwanda
Source: PLoS One. 2021 Dec 31;16(12):e0261744. doi: 10.1371/journal.pone.0261744 (PMC8719752; doi:10.1371/journal.pone.0261744)
Supplement: Attachment — Submitted filename: Response to Reviewers.docx [file pone.0261744.s001.docx]

**December 3^rd^, 2021**

**To: Sanjay Kumar Singh Patel, Ph.D.**

**Editor, PLoS One**

**Re: Re-submission for the manuscript PONE-D-21-30529**

Dear Dr. Patel,

On behalf of co-authors, I am writing to you to re-submit the revised version of our manuscript, which is entitled “Baseline knowledge and attitudes of COVID-19 among hotels’ staff: a cross-sectional study in Kigali, Rwanda”. We would like to thank you and the esteemed reviewers for the comments that we did gladly incorporate and address in the enclosed reply point-by-point letter.

We hope that you and the esteemed reviewers will find the updated manuscript as a good fit for the acceptance in PLoS One.

With kind regards,

Dr. Aphrodis Hagabimana

Rwanda Field Epidemiology and Laboratory Training Program,

Department of Epidemiology and Biostatistics,

University of Rwanda, Kigali, Rwanda

**Reviewer #1:**

**Hotels and other pandemic establishments are associated with higher transmission of coronavirus. So, better sensitization of staff and strengthening is required to control the coronavirus pandemic. In this paper, the authors investigate the baseline knowledge and attitudes on COVID-19 among hotels’ staff in Kigali, Rwanda: a cross-sectional study is performed using structured questionnaires to 104 participants. Although the number of participants is small, it provides an essential understanding of the COVID-19 pandemic.**

**Authors ‘reply:** We would like to thank Reviewer 1 for the overall summary and appreciation of the study in terms of an essential understanding of the COVID-19 pandemic.

**Minor comments:**

1) **Please discuss how the results stated in the manuscript are significant with only 104 hotel staff. Also, state the reason why more samples are not included in the manuscript.**

**Authors’ reply**: To the best of our knowledge, this was the first study that was conducted in the domain of hotel industry in urban area of a low-income country. We believe that this study provided important insights about the infection and control measures for COVID-19 in the hospitality sector. However, there were some limitations, which prevented us to conduct a larger survey among hotels’ staff in Kigali and other parts of the country. These limitations were related to the lack of study funding and movements’ restrictions during the time of the outbreak of COVID-19 pandemic. However, even though our findings may not provide a countrywide picture regarding the infection prevention and control measures for COVID-19 among hotels’ staff, we do believe that they provided some illumination on the importance of a systematic approach in consolidating evidence needed to identify priority populations for targeted intervention.

**2) The manuscript is well written. However, there are typos in the manuscript, such as fetal should be correct to fatal. Please go through the manuscript and correct all spelling errors.**

**Authors’ reply**: Thank you for pointing this out. We have revised the manuscript and addressed typos errors, as suggested.

**3) In the discussion section, please discuss how present studies is relevant in the present and future pandemic**.

**Authors’ reply**: Thank you for pointing this out. We have revised the discussion, as suggested.

**4) There are some formatting errors in the reference section. Please correct them.**

**Authors’ reply**: Thank you for pointing this out. We have corrected errors in the reference section.

**Reviewer #2:**

**The manuscript by Hagabimana et al. “Baseline knowledge and attitudes of COVID-19 among hotel staff. A cross sectional study in Kigali, Rwanda.” requires revision to address major concerns.**

**Authors’ reply:** We thank the esteemed Reviewer 2 for considering our paper and we hope you will find our updated version meeting your expectations.

**Comments and answers:**

**1. The manuscript may be polished extensively for the English language.**

**Authors’ reply:** Thank you for pointing this out. In the revised version of the manuscript, we have addressed the English language.

**2. Introduction (first paragraph): The information about mortality rate and various prevention approaches should be provided related to immunity and health i.e. doi: 10.1007/s12088-020-00908-0.**

**Authors’ reply**: We searched for the suggested reference, using the above doi, by the reviewer 2. The above doi did lead us to the reference: Rishi et.al.; Diet, Gut Microbiota and COVID-19; Indian J Microbiol; Sep 2020; <https://pubmed.ncbi.nlm.nih.gov/33012868/>. We feel this reference is out of scope in relation to our paper. We ask kindly reviewer 2 to confirm the reference.

**3. Introduction, the importance of this study may be more specifically highlighted and justified.**

**Authors’ reply**: Thank you for pointing this out. We have considered this comment in our revised manuscript.

**4. The author should provide at least one or two illustrations (additional Figures) to highlight the summary and significance.**

**Authors’ reply**: Thank you for pointing this out. This comment was addressed accordingly in the current version of the manuscript.
